# Supplementary material for: Effects of Trichoderma harzianum combined with Phanerochaete chrysosporium on lignin degradation and humification during chicken manure and rice husk composting
Source: Front Microbiol. 2025 Feb 28;16:1515931. doi: 10.3389/fmicb.2025.1515931 (PMC11906335; doi:10.3389/fmicb.2025.1515931)
Supplement: Supplementary file 1 [file Data_Sheet_1.zip › 6.Function/CK0_BFF/FunGuild/l1/BFF_vs_CK0_STAMP.pdf]

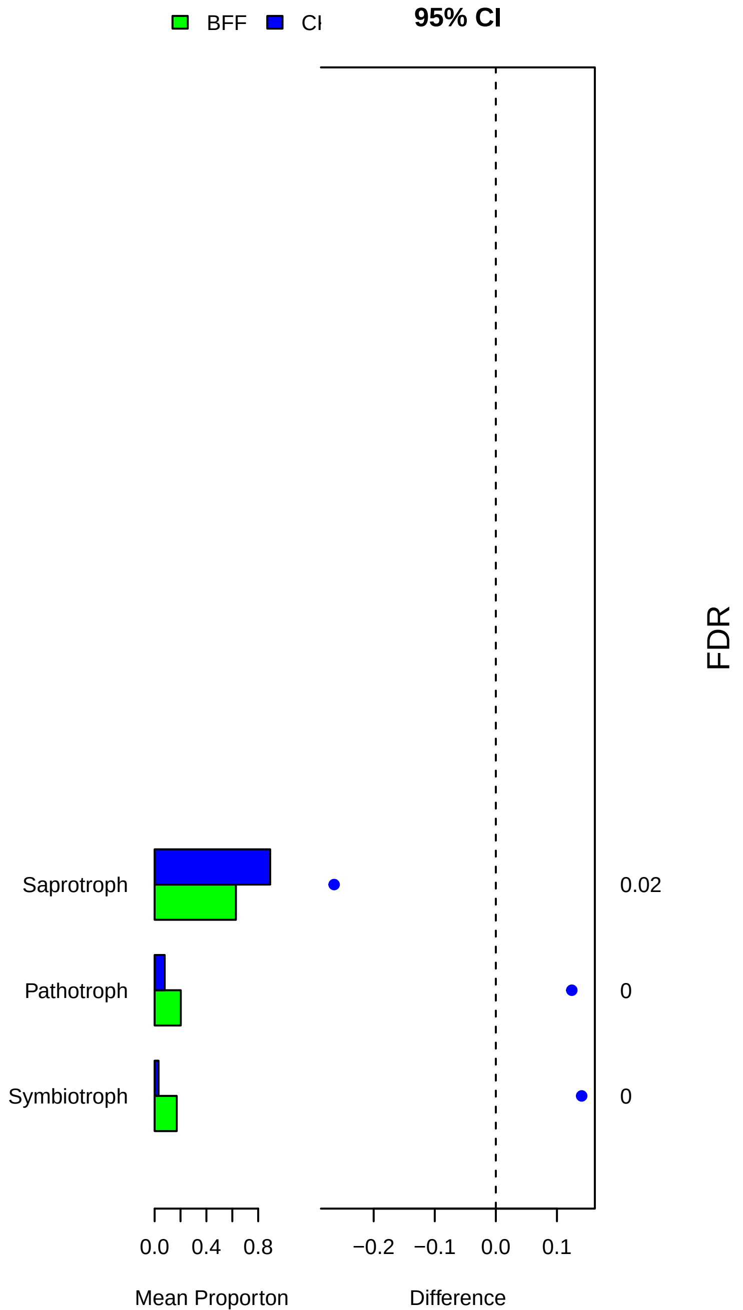
BFF
CI
95% CI
FDR
Saprotroph
Pathotroph
Symbiotroph
0.02
0
0
0.0
0.4
0.8
Mean Proporton
−0.2
−0.1
0.0
0.1
Difference
